# Associations Between Marijuana Use Trajectories and Educational and Occupational Success in Young Adulthood

**DOI:** 10.1007/s11121-018-0904-7

**Published:** 2018-04-28

**Authors:** Kara Thompson, Bonnie Leadbeater, Megan Ames, Gabriel J. Merrin

**Affiliations:** 1 Department of Psychology, St. Francis Xavier University, 2323 Notre Dame Ave., Antigonish, NS B2G 2W5 Canada; 2 University of Victoria, Victoria, BC V8P 5C2 Canada

**Keywords:** Marijuana, Trajectories, Educational attainment, Occupational outcomes, Young adult

## Abstract

Adolescence and young adulthood is a critical stage when the economic foundations for life-long health are established. To date, there is little consensus as to whether marijuana use is associated with poor educational and occupational success in adulthood. We investigated associations between trajectories of marijuana use from ages 15 to 28 and multiple indicators of economic well-being in young adulthood including achievement levels (i.e., educational attainment and occupational prestige), work characteristics (i.e., full vs part-time employment, hours worked, annual income), financial strain (i.e., debt, trouble paying for necessities, delaying medical attention), and perceived workplace stress. Data were from the Victoria Healthy Youth Survey, a 10-year prospective study of a randomly recruited community sample of 662 youth (48% male; *M*_age_ = 15.5), followed biennially for six assessments. Models adjusted for baseline age, sex, SES, high school grades, heavy drinking, smoking, and internalizing and oppositional defiant disorder symptoms. Chronic users (our highest risk class) reported lower levels of educational attainment, lower occupational prestige, lower income, greater debt, and more difficulty paying for medical necessities in young adulthood compared to abstainers. Similarly, increasers also reported lower educational attainment, occupational prestige, and income. Decreasers, who had high early use but quit over time, showed resilience in economic well-being, performing similar to abstainers. Groups did not differ on employment status or perceived workplace stress. The findings indicate that early onset and persistent high or increasingly frequent use of marijuana in the transition from adolescent to young adulthood is associated with risks for achieving educational and occupational success, and subsequently health, in young adulthood.

**Electronic supplementary material:**

The online version of this article (10.1007/s11121-018-0904-7) contains supplementary material, which is available to authorized users.

## Associations Between Marijuana Use Trajectories and Educational and Occupational Success in Adulthood

North American adolescents and young adults are among the youngest and most frequent users of marijuana in the developed world. According to a UNICEF survey (2013), Canadian youth ages 11 to 15 years old are the highest users, with 28% of young people reporting marijuana use in the last year. The United States (US) ranked 5th, with 22% of youth reporting marijuana use in the last year. As the landscape of legislation around marijuana use rapidly shifts across North America, concerns about the short- and long-term effects of youth marijuana use on health and well-being is growing. Already, we have seen marked shifts in the prevalence of use and decreases in youth perceptions of risk in jurisdictions that have legalized, or are planning to legalize, recreational marijuana use (Cerdá et al. 2017; Kerr et al. 2017; McKiernan and Fleming 2017). Moreover, there is growing evidence that marijuana use is associated with a myriad of negative consequences for youth, including injuries and accidents, delinquency, cognitive difficulties, mental health problems, and substance use dependency (Duncan et al. 2015; Hall 2015; Lisdahl et al. 2013; Simons et al. 2012). However, there is little consensus as to whether marijuana use is associated with poor psychosocial outcomes, particularly educational and occupational success in adulthood (National Academies of Sciences, Engineering, and Medicine 2017). The current study investigates the associations between previously established trajectories of marijuana use from ages 15 to 28 and multiple indicators of economic well-being in young adulthood including achievement levels (i.e., educational attainment and occupational prestige), work characteristics (i.e., full- vs part-time employment, hours worked, annual income), financial strain (i.e., debt, trouble paying for necessities, delaying medical attention), and perceived workplace stress.

### Economic Well-Being and Health

Socioeconomic status (SES) is a key determinant of health (Matthews and Gallo 2011; Thoits 2010). Key indicators of SES, such as education level, income, and occupational prestige have been consistently linked to health status, with research showing disproportionately higher levels of mental and physical health problems, and morbidity and mortality among those with lower SES compared to those with higher SES (Galea et al. 2011; Hout 2012; Reiss 2013; Stringhini et al. 2017). Life course approaches suggest that the associations between SES and health develop early and accumulate over time (Matthews and Gallo 2011; Singh-Manoux et al. 2004).

Adolescence and young adulthood is a particularly critical period when the foundations for healthy lifestyles and economic well-being are established (i.e., education attainment, securing full-time employment) (Schulte and Hser 2013). However, not all young people navigate this critical period successfully. Unemployment rates among young adults (aged 15 to 24) in Canada and the US have remained high for the last two decades, currently sitting at 13 and 10% respectively (OECD 2017). Moreover, the pursuit of postsecondary education among lower SES youth is declining in the US (Ma et al. 2016). The economic well-being of young people has been further compromised by the high costs of tuition, growing student debt, the rise in short-term and part-time employment, low wages, and delayed financial and residential independence (Bureau of Labor Statistics 2017; Morissette et al. 2013; Morissette 2016). Excessive marijuana use may further disadvantage young people by diminishing cognitive functioning and motivation that impacts educational and occupational goals, facilitating engagement in social contexts which compromise academic achievement, or by creating health problems that are incompatible with educational and occupational success (Fergusson and Boden 2008; Scholes-Balog et al. 2016; Zhang et al. 2016).

### Marijuana Use and Educational and Occupational Outcomes

Health risk behaviors, such as substance use, are associated with SES and also contribute to socioeconomic inequalities in health outcomes. A meta-analysis by Lemstra et al. (2008) showed that low SES youth ages 10 to 15 were 22% more likely to engage in alcohol and marijuana use compared to higher SES youth. Moreover, research has estimated that between one-half and three-quarters of the association between SES and mortality is attributable to health risk behaviors (Nandi et al. 2014; Stringhini et al. 2017). Research suggests that youth who use marijuana frequently may be particularly disadvantaged in terms of acquiring the skills necessary for socioeconomic capital and well-being in adulthood. Higher levels of adolescent marijuana use are negatively associated educational attainment (Horwood et al. 2010; Macleod et al. 2004; Silins et al. 2014), employment (Hara et al. 2013), and income (Ringel et al. 2006). A recent integrative meta-analysis across three large longitudinal studies from Australia and New Zealand found a consistent dose-response relationship between frequency of adolescent marijuana use and rates of high school completion and levels of educational attainment in young adulthood, after adjusting for 53 covariates (Silins et al. 2014). Further, in a 25-year birth cohort study, Fergusson and Boden (2008) found that greater marijuana use in adolescence was associated with lower income, greater welfare dependence, unemployment, and degree attainment in young adulthood, after accounting for a range of confounding factors (e.g., family SES, family functioning, deviant peer affiliations, grades, comorbid mental health disorders, and substance use).

Person-centered approaches looking at divergent trajectories of marijuana use across adolescence and young adulthood in relation to educational and occupational outcomes have added to this body of literature. Studies suggest that “chronic” users, typically characterized by early onset and high, persistent use across adolescent and young adulthood, were more likely than abstainers to be unemployed in adulthood (Lee et al. 2015a; Fergusson and Boden 2008; Schulenberg et al. 2005; Zhang et al. 2016). Chronic users also showed lower work commitment and achievement (Brook et al. 2011, 2013), lower income (Epstein et al. 2015), reduced financial stability (Brook et al. 2013), greater welfare dependency (Fergusson and Boden 2008), and lower degree attainment (Ellickson et al. 2004; Epstein et al. 2015) compared to abstainers. Moreover, trajectories characterized by high and increasing use levels during the late adolescence and young adulthood period also show risk for poor economic well-being, similar to chronic users (Brook et al. 2013; Epstein et al. 2015; Lee et al. 2015a).

However, the evidence is mixed and there is a growing number of studies finding no differences between trajectory groups and only weak or non-significant associations between marijuana use and educational and occupational outcomes, particularly after accounting for covariates such as sex, parent education, alcohol and tobacco use, and mental health and behavioral problems (Lee et al. 2015b; McCaffrey et al. 2010; Mokrysz et al. 2016; Popovici and French 2014; Scholes-Balog et al. 2016; White et al. 2015). Moreover, compared to evidence of the association between marijuana use and other health outcomes (i.e., mental health, injury, driving), there is much less research on the association between marijuana use and psychosocial outcomes (National Academies of Sciences, Engineering, and Medicine 2017). In a recent summary of the literature, the National Academies of Sciences, Engineering, and Medicine (2017) concluded that, to date, there is limited evidence supporting an association between marijuana use and educational and occupational outcomes.

Past research is limited by frequent failures to include potential confounding variables, as well as, the inclusion of single indicators of academic or economic outcomes. Typically, studies include one of the following three indicators: educational achievement (Windle and Wiesner 2004), income, or employment/unemployment (Zhang et al. 2016). White et al. (2015) also included occupational prestige and Brook et al. (2013) included financial stability. The development of targeted health promotion and harm reduction messages aimed at mitigating the potential short- and long-term harms from marijuana use requires more accurate information about the association between specific patterns of marijuana use and key social determinants of health, namely, educational attainment and economic capital. Examining multiple facets of economic well-being simultaneously, while controlling for important confounding variables, can advance our understanding of how marijuana use may confer economic risk, as well as possible barriers to economic success faced by those with various high risk trajectories of marijuana use.

### The Current Study

**Fig. 1:**
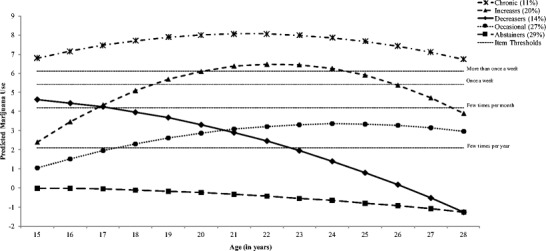
Plotted latent classes of marijuana use from 15 to 28 years of age. Note: Log-odd trajectories are on arbitrary scales; as such, the estimated thresholds that divide the categories of observed data are shown as dashed lines to facilitate interpretation. *Canadian Journal of Behavioural Science*, Volume 50, Issue 1, page # 21 Copyright © 2018 by the Canadian Psychological Association Inc. Reprinted by permission of the Canadian Psychological Association Inc.

In the current study, we build on previous literature and investigate the association between previously established trajectories of marijuana use from ages 15 to 28 (Thompson et al. 2018) and multiple indictors of economic well-being in young adulthood (ages 22 to 29). Past research with the current sample has identified five distinct marijuana use classes that include “abstainers” (*n* = 183; 29%) who never used marijuana; “occasional users” (*n* = 172; 27%) who had an onset of use in mid-late adolescence and used to a “few times a year” across young adulthood; “decreasers” (*n* = 89; 14%) who had high adolescent use (i.e., a few times per month at age 15) but steadily declined to less than a few times per year by age 23; “increasers” (*n* = 127; 20%) had an early onset and increased their use steeply across adolescence and young adulthood, peaking at more than once per week about age 22, and then gradually tapered their use to a few times per month by age 28; and “chronic users” (*n* = 69; 11%) who used marijuana more than once per week across all ages (see Fig. 1; Thompson et al. 2018).

Going beyond previous studies, we examine the associations between these trajectory groups and multiple indicators of economic well-being in young adulthood (ages 22 to 29) to investigate which specific aspects of economic well-being were more likely to be impacted negatively by marijuana use. We include commonly used indicators, such as educational attainment, employment status, and income, as well as less commonly used indicators of economic well-being, including occupational prestige, number of hours worked, indicators of financial strain (i.e., debt, trouble paying for basic necessities, delay of medical attention), and dimensions of perceived workplace stress (i.e., personal conflict, job instability, and workload demands). The models controlled for baseline SES, high school grades, adolescent levels of alcohol and tobacco use, and symptoms of internalizing disorders (i.e., anxiety and depression) and oppositional defiant disorder. Based on previous research, with single indicators of educational and economic outcomes, we expected that chronic users and increasers would report the poorer educational and occupational outcomes compared to other trajectories.

## Method

### Participants

Data from the Victoria Health Youth Survey (V-HYS) which collected data at six times across 10 years (between 2003 and 2013) was used in the current study (see Leadbeater et al. 2012for a detailed description). Among the 662 youth, most identified as Caucasian (85%), about half were male (48%), and the sample was representative of diverse socioeconomic groups. Attrition analyses showed youth who remained in the study (*N* = 478) were more likely to be female (*χ*^2^ (1, 662) = 8.77, *p* = .003) and had slightly higher T1 SES (parental occupational prestige; *M* = 6.73, *SD* = 1.66), *F*(1, 636) = 19.39, *p* < .001, compared to non-participants (*N* = 184; *M* = 6.05, *SD* = 1.94). No other group differences on study variables were significant.

### Procedure

Written consent of participation was obtained from youth and their parents or guardians (if the youth was under age 18) at each wave. A random sample of 9500 telephone listings was used to identify households (*n* = 1036) with an eligible (ages 12 to 18) youth. Among these households, 662 youth and their parents or guardians agreed to be a part of the study. Individual interviews were conducted by trained interviewers in the youth’s home or at another private location. Part of the survey assessment was self-administered to increase privacy and response rate. Gift certificates were given as incentives for participating at each wave. Retention rates were high across the six waves: 87% (T2; *n* = 578), 81% (T3; *n* = 539), 69% (T4; *n* = 459), 70% (T5; *n* = 463), and 72% (T6; *n* = 478). The university’s research ethics board approved the research protocol at each wave.

### Measures

#### Covariates

Youth self-reported their sex and age. Youth-reported parental occupation was recoded using the Hollingshead Occupational Status Scale (Hollingshead 1975; also see Bornstein et al. 2003) from 1 to 9. The highest level prestige from either parent was used as a proxy for socioeconomic status (SES). To assess high school grades (T1), youth were asked, “In general, what are your grades right now?” with response options ranging from 1 (*mostly F’s*) to 5 (*mostly A’s*). Those not currently in school were asked to report their most recent grades. Self-reported grades were highly correlated with obtained Grade 12 English grades, *r* = .62. Participants reported how often they had five or more drinks on one occasion in the past year to assess heavy episodic drinking (HED): 0 = *never*, 1 = *a few times a year*, 2 = *a few times a month,* 3 = *once a week*, and 4 = *more than once a week.* Smoking status was determined by the number of cigarettes participants reported consuming in the past week; dichotomized into 0 (*none*) and 1 (*one or more per week*). Oppositional defiant disorder (ODD) and internalizing symptoms were assessed with the Brief Child and Family Phone Interview (BCFPI; Cunningham et al. 2009). ODD symptoms (e.g., argue a lot with others? Are cranky?) was assessed with six items and depressive symptoms (e.g., feel hopeless? Have no interest in your usual activities?) and anxiety symptoms (e.g., worry about doing the wrong thing? Are overly anxious to please people?) were combined to create an internalizing symptoms subscale (12 items). Polychoric alphas (Gadermann et al. 2012) were good for each of the domains at T1 and T6, respectively: .79 and .85 for ODD and .86 and .93 for internalizing symptoms.

#### Marijuana Use

Youth were asked at each assessment, “How often did you use marijuana in the past 12 months?” Responses were coded on a five-point scale: 0 = *never*, 1 = *a few times a year*, 2 = *a few times a month,* 3 = *once a week*, and 4 = *more than once a week*. Quantity, the amount used in 1 day, was reported in response to the question: “during the last 3 months, on a day when you used marijuana, cannabis or hashish roughly how many joints did you usually have in that day?” (Count 10 puffs, 5 bong or pipe hits or ½ gram as equivalent to one joint)” (Zeisser et al. 2012).

#### Educational Attainment

At T6, youth reported their highest level of educational completed: high school or less (1); some postsecondary training (2); trade certificate or diploma (3); college or university certificate or diploma (4); and bachelor degree or higher (5).

#### Occupational Prestige

At T6, youth-reported job titles were recoded using the Hollingshead Occupational Status Scale (Hollingshead 1975; Bornstein et al. 2003). The nine categories are: (1) menial service workers (e.g., cleaner, housekeeper); (2) unskilled workers (e.g., cashier, server); (3) semi-skilled workers (e.g., childcare worker, roofer); (4) skilled workers/small business (e.g., administrative assistant, receptionist); (5) clerical and sales workers (e.g., book keeper, medical office assistant); (6) technicians/semi-professionals (e.g., lab technician, human resource officer); (7) managers/lesser professionals (e.g., teacher, social worker); (8) administrators/minor professionals (e.g., registered nurse, registered massage therapist); and (9) executives/major professionals (e.g., resident doctor, veterinarian).

#### Work Characteristics

Work characteristics were assessed using items developed for the V-HYS. Youth listed all jobs held since the last interview on a 24 month-by-month timeline (Desjardins and Leadbeater 2017; Leadbeater and Ames 2017). Youth provided additional information for each job held which were used to create the following work characteristic variables: (1) current employed status (i.e., not employed and employed part-time (0; < 30 h per week), employed full-time (1; ≥ 30 h per week)). Youth also reported the number of hours worked per week summed across jobs (range = 1 to 114.5 h). Youth reported total personal income from all sources (before taxes, including tips, commissions, scholarships, bursaries) for the previous fiscal year. Total income was recoded as (0 = *no income*, 1 = *$1 to $4,999*, 2 = *$5,000 to $9,999*, 3 = *$10,000 to $14,999*, …, 21 = *$100,000 or more*).

#### Financial Strain

At T6, financial stain was assessed using items developed for the V-HYS. Youth were asked how often they had *troubles paying for basic necessities* (one item; “How often do you have problems paying for basic necessities (i.e., food, clothing or rent)?” on a three-point scale: 0 = *never*, 1 = *sometimes*, 2 = *often*. Responses to *sometimes* and *often* were combined. Youth were also asked whether they had put off or *delayed medical attention* (four items; e.g., “During the past 6 months, have you ever put off or delayed any of the following because of financial reasons?... Going to the dentist.” Youth answered no (0) or yes (1). Responses were summed (range = 0 to 4).

Youth also reported on their financial debt (Leadbeater and Ames 2017). Those who had any debt (36%; not including student loans), specified the amount of money they owed to the following sources: (1) line of credit; (2) major credit cards; (3) store credit cards; (4) bank loans; and (5) other (e.g., back taxes owed to the government). The dollar amounts for total debt (i.e., excluding student debt) were recoded on a five-point scale (0 = *no debt*, 1 = *$1 to $4,999*, 2 = *$5,000 to $9,999*, 3 = *$10,000 or more*) for descriptive purposes. Debt was dichotomized to no debt (0) and any debt (1) for the final models.

#### Perceived Workplace Stress

Perceived workplace stress was assessed using 12 items derived from indicators included in the 2008 Stress in America Survey (American Psychological Association 2008). The perceived workplace stress scale has three subscales (Leadbeater and Ames 2017): *personal conflict* (five items; e.g., “problems with my supervisor”), *job instability* (three items; “job insecurity”), and *workload demands* (four items; e.g., “too heavy a workload”). Youth rated how significant each item impacted their stress level at work on a 4-point Likert scale (0 = *not at all significant* to 3 = *very significant*). Polychoric alphas were high for personal conflict (.81) and workload demands (.76) and adequate for job instability (.61; possibly reflecting the reduced number of items in this scale).

### Analytical Plan

Previously identified latent class trajectories of marijuana use from ages 15 to 28 for this sample were used: abstainers (29%), occasional users (27%), decreasers (14%), increasers (20%), and chronic users (11%) (see Thompson et al. 2018). Latent class growth analysis (LCGA; Jung and Wickrama 2008) was used to differentiate the marijuana use trajectories based on the frequency of marijuana use and time was represented by age in years, i.e., we restructured the six waves of data according to participant age. Following the three-step approach in Mplus version 7.3 (Muthén and Muthén 1998-2012) that statistically adjusts for the uncertainty in trajectory class membership, we examined differences between marijuana trajectory classes on indicators of educational and occupational functioning in young adulthood (ages 22 to 29; Asparouhov and Muthén 2014). Specifically, using multivariate regression, we examined differences between trajectory classes on multiple indicators of educational and occupational outcomes that included (1) achievement (i.e., educational attainment and occupational prestige), (2) work characteristics (i.e., employment status and number of hours worked per week), (3) income, (4) financial strain (i.e., debt, trouble paying for basic necessities, and delaying medical attention), and (5) perceived workplace stress (i.e., personal conflict, job instability, and workload demands). The five groups of academic and occupational outcomes (i.e., achievement, work characteristics, income, financial strain, and perceived workplace stress) were examined in separate models. All models adjusted for sex, baseline SES, heterogeneity in age, and youth-reported baseline levels (ages 12–18) of grades, alcohol and tobacco use, and symptoms of internalizing and oppositional defiance disorder. For variables with significant overall Wald *χ*^2^ statistics (*p* < .05), all possible pairwise comparisons between trajectory classes were tested. Preliminary analyses (e.g., ANOVA, chi-square distributions) were computed using SPSS version 23.

To account for missing data, models were fit using a full-information maximum likelihood (FIML) which allows individuals to contribute any information they have available to the likelihood function without the need to remove them from the analysis for having missing data. A robust maximum likelihood estimator was used to address any non-normality by adjusting the standard errors (MLR; Muthén and Muthén 1998-2012).

## Results

Means (or frequencies) and standard deviations (or percent) of T1 (ages 12–18) covariates and T6 (ages 22–29) educational and occupational functioning by marijuana trajectory class are presented in Table 1. As shown in previous research with the sample (Thompson et al. 2018), male participants were over-represented in the increasers and chronic user classes. Increasers and chronic users also reported lower levels of SES than youth in other trajectory classes. Abstainers and occasional users reported higher high school grades compared to youth in the other marijuana trajectory classes. Chronic users were more likely to smoke cigarettes compared to abstainers, occasional users, and increasers. Further, chronic users reported higher rates of ODD symptoms compared to abstainers, occasional users, and increasers.

**Table 1:** Descriptive statistics (means and standard deviations, frequencies, and percentages) of covariates and academic and occupational functioning by marijuana use trajectory class

	1. Abstainers (*n* = 183; 29%)	2. Occasional (*n* = 172; 27%)	3. Decreasers (*n* = 89; 14%)	4. Increasers (*n* = 127; 20%)	5. Chronic (*n* = 69; 11%)
	Mean (SD) or *n* (%)	Mean (SD) or *n* (%)	Mean (SD) or *n* (%)	Mean (SD) or *n* (%)	Mean (SD) or *n* (%)
Characteristics of trajectories					
Age of marijuana onset	17.07 (2.68)	16.56 (2.53)	14.37 (1.76)	15.11 (1.65)	13.28 (1.98)
Frequency of use at T6 (ages 22–29)					
Never	130 (95%)	28 (21%)	49 (78%)	6 (6%)	1 (2%)
A few times per year	7 (5%)	70 (53%)	13 (21%)	23 (25%)	3 (7%)
A few times per month	0 (0%)	21 (16%)	1 (2%)	16 (17%)	3 (7%)
Once a week	0 (0%)	5 (4%)	0 (0%)	13 (14%)	6 (13%)
More than once a week	0 (0%)	9 (7%)	0 (0%)	35 (38%)	32 (71%)
Quantity of use at T6 (ages 22–29)	0.04 (0.23)	0.55 (0.73)	0.17 (0.40)	1.27 (1.12)	2.60 (2.33)
T1 covariates (ages 12–18)					
Sex					
Male	74 (40%)	72 (42%)	42 (47%)	78 (61%)	41 (59%)
Female	109 (60%)	100 (58%)	47 (53%)	49 (39%)	28 (41%)
SES	6.66 (1.69)	6.75 (1.63)	6.64 (1.76)	6.56 (1.82)	6.00 (1.85)
Age	15.09 (1.91)	14.97 (1.89)	15.12 (2.06)	15.28 (1.79)	15.10 (1.87)
Course grades	3.25 (.76)	3.22 (.69)	2.91 (.83)	2.89 (.78)	2.59 (.80)
Heavy episodic drinking	0.16 (.53)	0.46 (.84)	0.80 (1.01)	0.97 (1.18)	1.39 (1.32)
Oppositional defiant disorder symptoms	3.83 (2.22)	4.01 (2.26)	4.69 (2.23)	4.16 (2.05)	5.88 (2.38)
Smoking status	7 (4%)	10 (6%)	14 (16%)	17 (13%)	26 (38%)
Internalizing symptoms	8.22 (4.27)	8.98 (4.23)	8.98 (4.26)	8.12 (4.16)	9.41 (3.92)
T6 young adulthood academic and occupational achievement (ages 22–29)					
Achievement					
Educational attainment (mean)	3.93 (1.43)	3.71 (1.47)	3.29 (1.44)	3.06 (1.35)	2.43 (1.41)
High school or less-1	14 (10%)	13 (10%)	9 (14%)	12 (13%)	16 (35%)
Some postsecondary-2	16 (12%)	26 (19%)	14 (21%)	26 (28%)	11 (24%)
Trade certificate or diploma-3	12 (9%)	13 (10%)	12 (18%)	20 (21%)	8 (17%)
College or university certificate or diploma-4	19 (14%)	18 (13%)	11 (17%)	16 (17%)	5 (11%)
Bachelor degree or higher-5	76 (56%)	65 (48%)	20 (30%)	20 (21%)	6 (13%)
Occupational prestige^a^ (Mean)	5.38 (1.81)	5.03 (1.97)	5.14 (2.01)	4.33 (1.78)	3.93 (1.59)
Menial service workers-1	1 (1%)	0 (0%)	1 (2%)	0 (0%)	1 (1%)
Unskilled workers-2	8 (6%)	21 (16%)	8 (12%)	16 (18%)	12 (27%)
Semi-skilled workers-3	11 (9%)	5 (4%)	4 (6%)	11 (12%)	3 (7%)
Skilled workers/small business-4	23 (18%)	29 (22%)	15 (23%)	36 (40%)	18 (41%)
Clerical and sales workers-5	16 (13%)	20 (15%)	4 (6%)	4 (4%)	2 (5%)
Technicians/semi- professionals-6	31 (25%)	28 (21%)	16 (25%)	10 (11%)	5 (11%)
Managers/lesser professionals-7	21 (17%)	13 (10%)	10 (15%)	8 (9%)	4 (9%)
Administrators/minor professionals-8	12 (10%)	8 (6%)	4 (6%)	5 (6%)	0 (0%)
Executives/major professionals-9	3 (2%)	7 (5%)	3 (5%)	1 (1%)	0 (0%)
Financial strain					
Total debt (not school debt)	$1483 ($3858)	$2271 ($8948)	$2466 ($4702)	$1883 ($5580)	$3000 ($6480)
$0	102 (75%)	83 (62%)	40 (61%)	59 (63%)	20 (44%)
$1–$4999	20 (15%)	32 (24%)	12 (18%)	21 (22%)	19 (41%)
$5000–$9999	9 (7%)	15 (11%)	7 (11%)	13 (14%)	2 (4%)
$10,000 or more	6 (4%)	5 (4%)	7 (11%)	1 (1%)	5 (11%)
Trouble paying for basic necessities	22 (16%)	30 (22%)	16 (24%)	26 (28%)	14 (30%)
Delay of medical attention (mean)	0.28 (.65)	0.59 (.88)	0.47 (.85)	0.50 (.74)	0.74 (.98)
Delay dentist	21 (15%)	40 (30%)	17 (26%)	29 (31%)	18 (39%)
Delay doctor visits	3 (2%)	6 (4%)	4 (6%)	4 (4%)	3 (7%)
Delay filling prescription	8 (6%)	15 (11%)	4 (6%)	7 (7%)	6 (13%)
Delay mental health treatment	7 (5%)	18 (13%)	6 (9%)	6 (6%)	7 (15%)
Work characteristics					
Full-time employment status	88 (64%)	81 (60%)	47 (71%)	57 (61%)	29 (63%)
Hours work per week	37.05 (18.09)	36.49 (17.39)	39.34 (18.65)	36.16 (17.32)	35.09 (13.50)
Income					
Annual income	$36,788 ($29,868)	$34,313 ($25,351)	$44,950 ($38,709)	$34,231 ($38,811)	$30,635 ($23,547)
Perceived workplace stress					
Personal conflict	4.82 (3.35)	4.84 (3.23)	5.03 (3.34)	4.34 (3.15)	5.78 (3.10)
Job instability	2.76 (2.14)	3.28 (2.01)	2.89 (2.11)	2.66 (1.94)	3.16 (2.04)
Workload demands	3.93 (3.13)	3.77 (2.76)	4.34 (2.85)	3.54 (2.37)	3.96 (2.73)

Sample sizes for each trajectory group are based on class assignment using the posterior probability of group membership. Percentages refer to column totals within each marijuana use class. Monetary values are rounded to the nearest hundredth

^a^Category titles shortened based on Hollingshead’s (1975) classification system

### Marijuana Use Trajectory Differences in Educational and Occupational Functioning

Table 2 presents the results for marijuana use trajectory class differences for educational and occupational functioning in young adulthood (ages 22 to 29). Adjusted means (or probabilities) are presented that account for sex, SES, heterogeneity in age, high school grades, and baseline levels of HED, ODD, internalizing symptoms, and smoking status.

**Table 2:** Means and standard errors (adjusted for covariates and other variables in the model) of academic and occupational functioning outcomes by marijuana use trajectories in young adulthood (ages 22 to 29)

	1. Abstainers (*n* = 183; 29%)	2. Occasional (*n* = 172; 27%)	3. Decreasers (*n* = 89; 14%)	4. Increasers (*n* = 127; 20%)	5. Chronic (*n* = 69; 11%)	Overall Wald	Pairwise comparisons
	Adjusted mean (SE)	Adjusted mean (SE)	Adjusted mean (SE)	Adjusted mean (SE)	Adjusted mean (SE)	*χ* ^2^	*p* < .05
Achievement							
Educational attainment	− .12 (.40)	− .21 (.41)	− .71 (.45)	− .73 (.39)	− 1.05 (.42)	17.60***	4, 5 < 1, 2
Occupational prestige	2.31 (.56)	1.96 (.55)	2.36 (.59)	1.25 (.49)	1.33 (.55)	20.02***	4, 5 < 1, 3; 4 < 2
Work characteristics							
Full-time (Pr)	.72	.62	.95	.58	.73	4.13	
Hours worked per week	44.16 (6.18)	38.88 (5.75)	54.85 (6.57)	37.26 (6.53)	41.08 (5.71)	12.63*	3 > 2, 4, 5
Income							
Annual income	6.58 (1.43)	6.22 (1.40)	8.35 (1.61)	4.68 (1.37)	5.00 (1.47)	15.16**	4, 5 < 3; 4 < 1, 2
Financial strain							
Any debt (not school debt) (Pr)	.28	.50	.50	.42	.65	10.69*	2, 3, 5 > 1
Trouble paying for basic necessities (Pr)	.34	.57	.42	.61	.66	8.63	
Delay of medical attention	.27 (.26)	.77 (.35)	.37 (.26)	.56 (.28)	.89 (.37)	22.29***	2, 5 > 1
Perceived workplace stress							
Personal conflict	2.85 (1.06)	2.94 (1.11)	3.00 (1.22)	2.35 (1.06)	3.83 (1.13)	3.92	
Job instability	3.00 (.63)	3.66 (.67)	3.13 (.70)	2.98 (.61)	3.31 (.64)	4.56	
Workload demands	3.46 (.85)	3.29 (.92)	3.83 (.96)	3.02 (.84)	3.37 (.92)	2.35	

All models control for sex, SES, T1 age, and T1 course grades, T1 heavy episodic drinking, T1 oppositional defiant disorder symptoms, T1 smoking status, and T1 internalizing symptoms. Each of the five academic and occupational sub-sections were analyzed in separate models

*p < .05, **p < .01, ***p < .001

#### Achievement

The omnibus test was significant for educational attainment (Wald *χ*^2^ = 17.60, *p* = .002). Post hoc pairwise comparisons showed that increasers and chronic users reported lower levels of educational attainment than abstainers and occasional users. Similarly, the omnibus test for occupational prestige was significant (Wald χ^2^ = 20.02, *p* < .001). Increasers and chronic users had lower levels of occupational prestige than abstainers and decreasers, and increasers had lower levels of occupational prestige than occasional users.

#### Work Characteristics

Classes did not differ on full-time employment status. However, the omnibus test for number hours worked per week was significant (Wald *χ*^2^ = 12.63, *p* = .013) and indicated that the decreasers reported working more hours per week than occasional users, increasers, and chronic users.

#### Income

Trajectory class differences for income were significant (Wald *χ*^2^ = 15.61, *p* = .004). Increasers and chronic users reported lower incomes than decreasers. Increasers also reported lower incomes than abstainers and occasional users.

#### Financial Strain

Occasional users, decreasers, and chronic users were more likely to have debt (not school-related) than abstainers (Wald *χ*^2^ = 10.69, *p* = .030). Marijuana use trajectory classes did not differ in their ability to pay for basic necessities. Trajectory class differences were significant for delay of medical attention (Wald *χ*^2^ = 22.29, *p* < .00) and indicated that occasional and chronic users were more likely to delay medical attention than abstainers.

#### Perceived Workplace Stress

There were no significant differences found between marijuana trajectory classes for personal conflict, job instability, or workload demands.

We also conducted a sensitivity analysis on our models, adjusting for T6 (concurrent) levels of alcohol use, tobacco use, symptoms of internalizing and symptoms of oppositional defiant disorder, rather than baseline levels. Adjusting for T6 covariates reduced our sample size considerably (*n* = 662 to *n* = 449) because Mplus excludes participants who do not have complete data on covariates. Despite this reduced sample size, the findings were largely robust (see the supplementary Table). Only the omnibus test for debt became non-significant (Wald *χ*^2^ = 10.69, *p* = .030). Minor changes were also observed in pairwise comparisons, such that additional significant differences in educational attainment between abstainers and decreasers emerged, as did a significant difference in income between abstainers and increasers and chronic users. The observed difference between in delayed medical attention became non-significant between abstainers and chronic users at *p* = 0.059.

## Discussion

This study examined the associations between patterns of marijuana use in youth across the transition from adolescence to young adulthood (i.e., ages 15 to 28) and multiple indicators of educational achievement and economic success in young adulthood (ages 22–28). Consistent with past research, the findings showed significant differences in educational attainment and occupational prestige between classes (Ellickson et al. 2004; Epstein et al. 2015), even after including confounding variables that could account for these differences (i.e., family SES, grades, substance use, internalizing symptoms and ODD symptoms). Both increasers and chronic users reported significantly lower educational attainment and occupational prestige. Youth in both groups were less likely to complete a bachelor degree compared to other classes. Most increasers worked as skilled labors (40%) or unskilled labors (18%). Similarly, chronic users also predominantly worked as skilled (41%) or unskilled labors (27%). However, notably, a significant proportion of youth did complete a trade certificate or received a college diploma (38% and 28% respectively) by young adulthood.

Given the differences in educational qualifications and employment opportunities, it is not surprising that there were significant differences in earnings between these higher-use classes and abstainers and occasional users. Lower income can create difficulties paying for necessities and chronic users had more accrued credit card debt and were more likely to report delays in seeking medical attention for financial reasons than abstainers. A greater proportion of chronic users reported delaying visits to the dentist (39%), filling prescriptions (13%) and delaying mental health treatments (15%) due to financial difficulties compared to abstainers. Past research has consistently showed that chronic users have poor health outcomes in adulthood (Terry-McElrath et al. 2017). In previous research, chronic users also show higher levels of symptoms of depression and anxiety and as well as behavioral problems (i.e., attention deficit hyperactivity disorder symptoms, oppositional defiant symptoms, conduct symptoms) and substance use problems as young adults (Epstein et al. 2015; Thompson et al. 2018). Chronic users also report poorer physical health and more serious injuries, physical ailments (i.e., headaches, abdominal pain, backaches, and dizziness), and respiratory problems compared to lower-use classes (Ames and Leadbeater under review; Terry-McElrath et al. 2017). Despite having greater health needs, the findings of the current study suggest that the financial strain experienced by chronic users may result in reduced access to health care. This may sustain a negative cycle over time with lower income and financial strain exacerbating or contributing to poor health outcomes and health concerns interfering with future economic well-being by reducing work productivity and increasing absenteeism.

In contrast to previous literature, we found no differences between trajectory groups for employment status or average number of hours worked. Chronic and increasing use groups were as likely to be employed full-time as abstainers and occasional users. Recent longitudinal studies found higher levels of unemployment among the heaviest using classes in the mid-thirties and early forties (Lee et al. 2015a; Terry-McElrath et al. 2017; Zhang et al. 2016). Therefore, we might expect that continued use across adulthood and evidence of accumulating health problems experienced by chronic users may result in future challenges for employment stability and performance that are not yet evident in young adulthood. There were also no differences between classes in perceived workplace stress (i.e., personal conflict, job instability, or workload demands). Previous work with this sample found that higher levels of adolescent ODD symptoms were associated with higher levels of perceived personal conflict and job instability (Leadbeater and Ames 2017) and chronic users showed high adolescent levels of ODD symptoms (Thompson et al. 2018). Thus, workplace challenges may be more directly linked to co-occurring behavioral problems, such as ODD symptoms, rather than marijuana use per se. Associations between marijuana use and workplace stress may not have been significant due to controlling for ODD symptoms and other confounding factors in the current study. Further, our perceived workplace stress is self-reported in the current study. To better understand the association between work performance (i.e., personal conflict) and marijuana use, future studies should include employer-rated performance indicators.

Despite their profile of early high use of marijuana in adolescence and evidence of similar adolescent risks as chronic users (i.e., ODD symptoms, heavy drinking; Thompson et al. 2018), *decreasers*, who use marijuana only a few times a month at age 15 and decrease to no use or only infrequent use by early adulthood, show resilience in terms of economic well-being during young adulthood. Their level of educational attainment and occupational prestige was statistically similar to that of abstainers and occasional users, but the types of education and occupations they pursued differed. Decreasers were more likely than abstainers to pursue trade certificates and also largely worked as technicians (25%) or semi-skilled professionals (23%). Given these professional paths, decreasers also reported working more hours per week than other classes and had significantly higher earnings compared to increasers and chronic users. These findings are consistent with Lee et al. (2015a), who found that youth who use early but also quit early are not disadvantaged in terms of employment in adulthood. Overall, our findings further demonstrate that adolescence is a critical period for building the foundation for economic well-being. Heavy marijuana use during adolescence and across the transition to adulthood is clearly related to the educational and occupational success that supports health and well-being for some youth.

In the context of current findings and the growing body of literature on the health and social impacts of marijuana use, it is becoming clear that marijuana use does not occur in isolation from other risk factors and that risks for both early heavy marijuana use and poor economic well-being begin early (Marmorstein and Iacono 2011). The likely cascading mechanisms that link marijuana and educational and economic risks are not clear. However, early high frequency use, as well as the predictors of this early high use (i.e., low SES) and the co-occurring behavioral problems that typically accompany heavy adolescent use (i.e., ADHD, ODD symptoms, conduct symptoms and heavy drinking), together may undermine educational achievement and subsequent economic success. These effects could be either direct, by reducing motivation (Ringel et al. 2006) and impacting cognitive functioning and learning ability (National Academies of Sciences, Engineering, and Medicine 2017), or indirect, by increasing the likelihood of involvement with delinquent peers and activities. Educational achievement is the gateway to future occupational opportunities and postsecondary credentials are directly linked to employment and income levels (Ma et al. 2016). Inadequate educational credentials and training may have a cascading effect other indicators of economic well-being throughout adulthood. Thus, delaying use onset and encouraging more moderate or occasional use patterns, so as to minimize disruptions to educational goals, will be critical to shifting the observed patterns. Persistent high frequent use of marijuana beyond adolescence may also further impede economic success due to increasing health concerns, lack of motivation, and loss of productivity.

### Limitations

Limiting the generalizability of the findings, our sample is Canadian and predominately Caucasian. While similar trajectory classes have been identified in samples from the US, this sample has a higher proportion of youth classified into a chronic or increaser class and the frequency of use among these high-risk classes was higher compared to most US-based community samples (Brook et al. 2011; Thompson et al. 2018). Studies with youth with lower frequency use may be less likely to replicate our findings. The findings may also not generalize to youth from large multi-ethnic cities or minority populations. Moreover, as legalization of recreational marijuana is implemented, we may see shifts in acceptance of use and observed use patterns in youth (Salas-Wright et al. 2015). All measures were self-report; thus, reports of substance use may be underreported. Also, while we controlled for multiple potential confounds, it is possible that there are other explanatory mechanisms that have not been accounted for the in the current study. To better understand how marijuana use and its associated outcomes unfold and grow over time, future research should employ a dynamic cascade model to elucidate the transactional relationships between marijuana use and other co-occurring health problems and identify opportunities to disrupt or redirect these developmental pathways (Dodge et al. 2009).

### Conclusions

Our findings suggest that patterns of marijuana use characterized by early onset and high or increasingly persistent use across young adulthood present risks for educational and occupational success in young adulthood. This study is unique in assessing multiple indicators of educational and economic outcomes and in controlling for multiple potential confounds that could explain the links between trajectories of use and outcomes. Chronic users and increasers, reported lower levels of educational attainment, lower occupational prestige, lower income, greater debt, and more difficulty paying for medical necessities. The economic disadvantages already experienced by these youths may also affect their capacity to access health care and contribute to physical and mental health problems associated with chronic marijuana use. The findings add to the growing body of literature elucidating the cumulative impact of the early persistent high use or increasing use of marijuana and suggest that patterns of use negatively impact economic well-being in young adulthood.

## Electronic Supplementary Material

ESM 1 (DOCX 16 kb)
