# Supplementary material for: Associations Between Marijuana Use Trajectories and Educational and Occupational Success in Young Adulthood
Source: Prev Sci. 2018 Apr 28;20(2):257–69. doi: 10.1007/s11121-018-0904-7 (PMC6414467; doi:10.1007/s11121-018-0904-7)
Supplement: Supplementary file 1 — (DOCX 16 kb) [file 11121_2018_904_MOESM1_ESM.docx]

Supplemental Table Means and standard errors (adjusted for T1 and T6 covariates and other variables in the model) of academic and occupational functioning outcomes by marijuana use trajectories in young adulthood (ages 22 to 29)

|  | 1. Abstainers  (*n* = 183; 29%) | 2. Occasional  (*n* = 172; 27%) | 3. Decreasers  (*n* = 89; 14%) | 4. Increasers  (*n* = 127; 20%) | 5.  Chronic  (*n* = 69; 11%) | Overall Wald | Pairwise  Comparisons |
| --- | --- | --- | --- | --- | --- | --- | --- |
|  | Adjusted  mean (SE) | Adjusted mean (SE) | Adjusted  mean (SE) | Adjusted mean (SE) | Adjusted mean (SE) | χ^2^ | *p* < .05 |
| *Achievement* |  |  |  |  |  |  |  |
| Educational attainment | .28 (.39) | .17 (.39) | -.45 (.43) | -.46 (.38) | -.55 (.45) | 17.51*** | 4, 5 < 1, 2; 3 < 1 |
| Occupational prestige | 2.51 (.55) | 2.16 (.56) | 2.49 (.58) | 1.46 (.51) | 1.61 (.57) | 16.39** | 4, 5 < 1, 3; 4 < 2 |
|  |  |  |  |  |  |  |  |
| *Work characteristics* |  |  |  |  |  |  |  |
| Full-time (*Pr*) | .83 | .72 | .97 | .57 | .80 | 5.42 |  |
| Hours work per week | 43.03 (6.07) | 38.03 (5.68) | 53.81 (6.27) | 33.03 (7.62) | 40.00 (5.56) | 13.19* | 3 > 2, 4, 5 |
|  |  |  |  |  |  |  |  |
| Annual income^a^ | 8.12 (1.43) | 7.59 (1.42) | 9.33 (1.54) | 5.57 (1.36) | 6.19 (1.51) | 19.47** | 4, 5 < 1, 3; 4 < 2 |
|  |  |  |  |  |  |  |  |
| *Financial Strain* |  |  |  |  |  |  |  |
| Any debt (not school debt) (*Pr*) | .24 | .36 | .37 | .25 | .47 | 4.69 |  |
| Trouble paying for basic necessities (*Pr*) | .21 | .34 | .30 | .40 | .29 | 3.28 |  |
| Delay of medical attention | .16 (.26) | .55 (.33) | .35 (.25) | .50 (.26) | .63 (.35) | 19.14*** | 2, 4 > 1 |
|  |  |  |  |  |  |  |  |
| *Perceived workplace stress* |  |  |  |  |  |  |  |
| Personal conflict | 2.15 (1.04) | 2.01 (1.06) | 2.56 (1.17) | 1.80 (1.05) | 2.58 (1.04) | 1.74 |  |
| Job instability | 2.61 (.59) | 3.06 (.64) | 2.86 (.65) | 2.65 (.59) | 2.74 (.65) | 2.36 |  |
| Workload demands | 3.05 (.88) | 2.69 (.97) | 3.59 (.96) | 2.70 (.89) | 2.64 (1.00) | 2.71 |  |

*Note.* All models control for sex, SES, T1 age, and T1 course grades, T6 smoking status, T6 heavy episodic drinking, T6 oppositional defiant disorder symptoms, and T6 internalizing symptoms. ^a^Separate model.

**p* < .05, ***p* < .01, ****p* < .001.
